# SEMA3A in Proliferative Vitreoretinopathy: Exogenous Activity, Endogenous Regulation, and the MicroRNA Hypothesis

**DOI:** 10.3390/biomedicines14102186

**Published:** 2026-09-27

**Authors:** Bohao Wang, Siying Li, Yu Cao, Aimin Sun, Muzi Li, Jinfeng Qu

**Affiliations:** 1 Department of Ophthalmology, Peking University People’s Hospital, Beijing 100044, China; 2411110456@stu.pku.edu.cn (B.W.);; 2 Beijing Key Laboratory of Ocular Disease and Optometry Science, Peking University People’s Hospital, Beijing 100044, China

**Keywords:** proliferative vitreoretinopathy, retinal pigment epithelium, epithelial plasticity, epithelial–mesenchymal transition, microRNA, SEMA3A, ocular-fluid biomarkers, miRNA therapeutics, molecular staging, precision medicine

## Abstract

Proliferative vitreoretinopathy (PVR) remains a leading cause of surgical failure after rhegmatogenous retinal detachment (RRD) repair. Although PVR involves multiple cell types and signaling pathways, epithelial–mesenchymal transition (EMT) and related plasticity of retinal pigment epithelial (RPE) cells are major contributors to fibrocellular membrane formation and contraction. Semaphorin 3A (SEMA3A) has therefore attracted interest as a potential negative regulator of these pathogenic RPE responses. Recombinant SEMA3A inhibits RPE migration and proliferation and attenuates experimental PVR when administered at model induction. Yet this benefit does not establish that endogenous *SEMA3A* transcript levels are reduced. Published Source Data from a mouse model of traumatic PVR show significantly increased endogenous *Sema3a* transcript levels in RPE preparations compared with controls. The question is therefore why PVR progresses despite increased endogenous *Sema3a* transcript levels in this model. Possible explanations include microRNA-mediated translational repression, altered protein processing or secretion, reduced extracellular availability, and impaired NRP1/plexin signaling. MicroRNAs (miRNAs) are particularly relevant because they can reduce protein output without a parallel decline in mRNA and can regulate multiple features of RPE EMT. This narrative review examines current evidence for SEMA3A in PVR, summarizes miRNA regulation of RPE EMT and related PVR phenotypes, and evaluates direct and indirect evidence for miRNA–SEMA3A interactions. We also compare the miRNA hypothesis with alternative explanations for inadequate endogenous SEMA3A activity and consider its implications for ocular biomarkers and target-site-selective RNA therapy. Current evidence makes miRNA-mediated regulation of SEMA3A a plausible and testable mechanism, although a causal role has yet to be demonstrated in RPE cells or PVR.

## 1. Introduction

Proliferative vitreoretinopathy (PVR) remains a leading cause of failure after rhegmatogenous retinal detachment (RRD) repair and may also complicate ocular trauma or intraocular surgery. It represents an aberrant wound-healing response in which inflammation, cell migration and proliferation, extracellular matrix (ECM) deposition, and tissue contraction culminate in epiretinal, subretinal, and intraretinal fibrosis. The resulting traction can cause recurrent retinal detachment and limit visual recovery despite anatomically successful surgery [1,2,3]. Surgery remains the mainstay of treatment. Although corticosteroids, antimetabolites, retinoids, biologics, and other pharmacological adjuncts have been evaluated, none has shown sufficiently consistent benefit to enter clinical treatment [4,5,6,7]. This lack of clinical progress likely reflects the cellular and molecular heterogeneity of PVR, together with uncertainty over which pathway should be targeted and when treatment is most likely to be effective.

PVR is now recognized as a multicellular form of retinal fibrosis. Analyses of human membranes, together with single-cell and lineage-resolved experimental studies, have identified RPE, myeloid, Müller glial, and fibroblast-like populations that contribute to inflammation, matrix remodeling, and tissue contraction; among these populations, RPE cells make a central contribution to membrane formation [8,9,10,11]. Following a retinal break or detachment, they are displaced from their polarized monolayer and exposed to vitreous growth factors, transforming growth factor-β (TGF-β), inflammatory mediators, serum components, and abnormal mechanical cues. These stimuli promote the loss of cell–cell junctions, polarity, and other features of differentiated RPE, while inducing migratory, proliferative, matrix-producing, and contractile properties. These changes are generally described as epithelial–mesenchymal transition (EMT) and contribute directly to the formation and contraction of PVR membranes [12,13,14]. Single-cell and lineage-tracing studies indicate, however, that RPE cells occupy a continuum of intermediate states rather than undergoing a uniform and complete transition. We therefore use RPE EMT as a practical term encompassing both partial and more advanced EMT-like states.

SEMA3A is relevant to this process because it can restrain several of the RPE responses that promote PVR. Bai and colleagues showed that recombinant SEMA3A inhibited RPE migration and vascular endothelial growth factor (VEGF)-driven proliferation and reduced disease severity in a rabbit PVR model when co-administered at model induction [15]. This study provided proof of principle that exogenous SEMA3A can suppress PVR-related RPE behavior and attenuate experimental disease. It did not, however, test the treatment of established PVR or establish endogenous SEMA3A deficiency. Transcriptomic observations further complicate a simple deficiency model. In the mouse traumatic PVR study, the authors’ published Source Data show significantly increased endogenous *Sema3a* transcript levels in RPE preparations compared with controls [10]. In the human membrane dataset, *SEMA3A* transcript levels did not differ significantly between PVR membranes and internal limiting membrane specimens [8]. The question is therefore not simply whether SEMA3A expression declines, but why PVR progresses despite increased endogenous *Sema3a* transcript levels in the mouse model. Transcript abundance alone cannot determine how much biologically active SEMA3A is produced, released, and delivered to responsive cells at the relevant stage of disease. The defect could lie in post-transcriptional regulation, protein processing or secretion, extracellular sequestration or degradation, or signaling through neuropilin-1 (NRP1) and plexin receptors.

Among these possibilities, microRNAs (miRNAs) deserve particular attention. miRNAs can reduce protein production without a parallel decrease in mRNA abundance. They can also regulate several components of RPE EMT within the same cellular state, making them plausible links between altered SEMA3A activity and the wider phenotypic changes that drive PVR. This review places the SEMA3A question within the cellular and temporal biology of PVR, with particular emphasis on RPE EMT. It critically examines the evidence for exogenous and endogenous SEMA3A, summarizes current knowledge of miRNA regulation in RPE EMT and PVR, and evaluates direct and indirect evidence for miRNA–SEMA3A interactions. By considering this hypothesis alongside defects in ligand processing, extracellular availability, and receptor signaling, we aim to clarify whether miRNA-mediated regulation can account for a possible mismatch between *SEMA3A* transcript abundance and biological activity, and what evidence would be required to translate this relationship into ocular biomarkers or target-site–selective miRNA therapy.

## 2. Scope and Approach

This focused narrative review draws on the literature identified through searches of PubMed/MEDLINE and Europe PMC from database inception through 31 August 2026. It was not designed as a systematic review. Search terms covered PVR, retinal detachment, RPE EMT and phenotypic plasticity, SEMA3A, NRP1/plexin signaling, miRNAs, extracellular vesicles, ocular-fluid biomarkers, and RNA-based therapies. Searches were supplemented by examining the reference lists of relevant primary studies and recent reviews. Because direct evidence linking miRNAs, SEMA3A, RPE EMT, and PVR is limited, we also considered studies from other tissues when they provided experimentally validated information on miRNA–SEMA3A interactions or TGF-β-responsive fibrotic mechanisms with clear relevance to RPE biology. Processed expression data and author-reported gene-level statistics were examined in the deposited GSE179603 analysis and the published Source Data accompanying the mouse GSE244811/GSE244812 study. No new differential-expression analysis was performed. Extracted values and dataset details are provided in Supplementary File S2. Search details and representative queries are provided in Supplementary File S1, and summaries of the included studies are provided in Supplementary File S2. The resulting literature was synthesized narratively around the mechanistic and translational questions defined above.

Priority was given to evidence from human PVR specimens, RPE-resolved experimental models, well-characterized human RPE systems, mechanistic miRNA–target studies with rescue experiments, and in vivo PVR endpoints. Throughout the review, computational predictions were distinguished from reporter-based and endogenous target validation; *SEMA3A* transcript abundance was distinguished from the production and availability of biologically active ligand; and prevention at disease induction was distinguished from treatment of established PVR. Associations between ocular-fluid miRNAs and disease were interpreted primarily as biomarker evidence unless supported by functional studies in relevant cells or tissues. We compared the mechanistic evidence across studies and considered how the findings relate to RPE EMT and PVR.; no formal evidence-grading system was applied.

## 3. PVR Pathobiology: From Retinal Injury to Contractile Fibrosis

### 3.1. From Retinal Injury to Fibrocellular Membrane Formation

PVR is initiated when a retinal break, retinal detachment, ocular trauma, or intraocular surgery disrupts the barriers that normally separate retinal cells from the vitreous and circulating factors. RPE cells can leave their monolayer and enter the vitreous cavity or subretinal space, while breakdown of the blood–retinal barrier permits serum proteins, platelets, leukocytes, and soluble mediators to reach retinal surfaces. The resulting wound-healing environment favors cell attachment, migration, and survival [1,2,3].

Fibrocellular membranes develop as displaced RPE cells, inflammatory cells, glia, and other responsive populations accumulate and deposit extracellular matrix (ECM). Fibronectin, collagens, and other matrix proteins provide a scaffold for further cell attachment and tissue remodeling. With time, some cells acquire myofibroblast-like features, including α-SMA expression and prominent actin stress fibers. Force generated by the actomyosin cytoskeleton is transmitted through the surrounding matrix, allowing epiretinal and subretinal membranes to contract. The resulting traction produces retinal wrinkling and fixed folds and may ultimately cause recurrent detachment [1,2,3].

Inflammation, proliferation, and contraction are often described as successive phases of PVR, but they overlap in practice. Inflammatory signaling may persist as extracellular matrix accumulates and fibrocellular membranes contract [2,3]. Their relative contribution nevertheless changes as the disease progresses. Reversing established PVR may be more difficult than preventing its development. Early intervention therefore deserves particular attention [5]. Clinical PVR grading records anatomical severity, including vitreous cells, retinal wrinkling, fixed folds, and tractional detachment, but does not identify the dominant cellular or molecular process in an individual eye [2,4]. Eyes with a similar anatomical grade may therefore differ in their underlying biological activity [3,4].

### 3.2. The Multicellular Fibrotic Microenvironment

RPE cells are prominent contributors to PVR, but they do not act alone. Retinal breaks give displaced RPE cells access to the vitreous and retinal surfaces, where they can migrate, proliferate, secrete matrix proteins, and acquire contractile properties. Resident microglia and recruited monocyte-derived macrophages supply inflammatory and profibrotic mediators. Müller glia undergo reactive changes and contribute additional cellular and paracrine support to the developing membrane. Hyalocytes and other vitreous- or circulation-derived cells may also participate. Several of these lineages can acquire overlapping fibroblast- or myofibroblast-like features, which helps explain the mixed cellular composition of mature PVR membranes [1,2,3,8].

These cells respond to a shared but changing pool of growth factors, cytokines, chemokines, and matrix-derived signals. TGF-β promotes junctional disassembly, ECM production, and contractile differentiation in RPE cells. PDGF, VEGF, EGF, and related pathways support cell survival, migration, and proliferation, while inflammatory cytokines and chemokines maintain leukocyte recruitment and modify RPE and glial behavior. Once matrix has accumulated, cell–matrix adhesion and actomyosin tension can further stabilize migratory and contractile states. The biological activity of a PVR membrane therefore depends on both its cellular composition and its stage of maturation [1,2,3,12,13,14].

Recent studies have provided greater cellular resolution. RNA sequencing and imaging mass cytometry of human PVR membranes identified RPE-lineage cells, immune cells, glia, and α-SMA-positive myofibroblast-like cells within the same lesions [8]. In a mouse PVR model, single-cell RNA sequencing combined with genetic lineage tracing showed that RPE-derived cells made the largest cellular contribution to fibrotic lesions and passed through metabolic, proliferative, and EMT-associated states. Immune and Müller glial cells also contributed to PVR-associated fibroblast populations [9]. Epigenomic and transcriptomic profiling of RPE cells isolated from traumatic PVR revealed widespread changes in chromatin accessibility and enhancer activity, including activation of RANK–NFATc1 and gene programs associated with migration, proliferation, ECM organization, and cytokine production [10].

RPE cells thus remain a major source of pathogenic behavior, but their state is shaped by interactions with other cells and by changes in the local environment. The same studies also show why bulk membrane RNA requires cautious interpretation. Transcript abundance in a whole membrane is influenced by the proportions and states of its constituent cells and cannot be assigned directly to a particular cellular source or signaling activity.

### 3.3. RPE EMT and the Development of Contractile Membranes

Among the cell populations involved in PVR, RPE cells are particularly important because they can provide several of the functions required for membrane formation. Once displaced from their monolayer, RPE cells lose junctional and polarity programs and acquire motility, proliferative capacity, matrix production, and contractility. These changes are conventionally described as RPE EMT [12,13,14]. Lineage-tracing studies provide direct support for earlier histological observations: RPE-derived cells are a major component of fibrotic lesions and constitute the largest cellular fraction in experimental PVR [8,9].

RPE EMT has direct consequences for membrane formation and traction. Migrating RPE cells populate epiretinal and subretinal surfaces, and cell-cycle re-entry increases the number of cells within the developing membrane. Fibronectin, collagen, and other ECM components form the fibrotic scaffold, while α-SMA-containing actomyosin structures generate contractile force. Altered secretion from RPE cells can also modify local inflammatory and glial responses. Nevertheless, the combination of motility, matrix production, and contractility makes RPE EMT a central mechanism of PVR, even though other cell populations also contribute. The cellular events leading to PVR membrane formation and contraction are illustrated in Figure 1.

SEMA3A is relevant because it acts on processes engaged early in RPE EMT. As a secreted guidance molecule, it regulates cytoskeletal organization and cell movement. Bai et al. showed that recombinant SEMA3A inhibited RPE migration and VEGF-responsive proliferation and reduced rabbit PVR when given at model induction [15]. These experiments show that selected RPE responses remain sensitive to added SEMA3A. They do not establish whether endogenous SEMA3A signaling becomes ineffective during EMT, nor did they examine ECM deposition, membrane contraction, or treatment of established PVR. The current evidence therefore demonstrates pharmacological responsiveness to exogenous SEMA3A but leaves the status of the endogenous pathway unresolved.

## 4. SEMA3A in PVR: Exogenous Activity and Endogenous Regulation

### 4.1. Biological Rationale for SEMA3A in RPE EMT

SEMA3A is a secreted class 3 semaphorin first described as a repulsive axon-guidance cue. Outside the developing nervous system, it regulates cell polarity, adhesion, and migration by coupling extracellular guidance signals to cytoskeletal remodeling. Most cellular responses to SEMA3A require a receptor complex containing neuropilin-1 (NRP1) and a class-A plexin. NRP1 participates in ligand recognition and receptor assembly, whereas the plexin provides much of the intracellular signaling capacity [16,17]. NRP1 also serves as a coreceptor for VEGF165. Although this shared receptor links the two pathways, SEMA3A and VEGF165 engage partly distinct receptor determinants and do not behave simply as competing ligands at a single binding site [16,17,18].

These functions intersect with the early stages of RPE EMT. Before contributing to a PVR membrane, displaced RPE cells lose junctional organization and polarity, reorganize the actin cytoskeleton, leave the monolayer, and migrate across retinal surfaces. Cell-cycle re-entry expands the number of cells available for membrane formation, whereas matrix deposition and actomyosin-dependent contraction become more prominent as fibrosis matures. SEMA3A is most directly relevant to the earlier migratory phase. In neuronal models, LIMK–cofilin and RhoA–ROCK–myosin II signaling contribute to SEMA3A-dependent changes in actin organization and directional movement [19,20]. These pathways have not been confirmed in RPE cells during PVR, although they provide plausible mechanisms for an effect on RPE motility. The expression of NRP1 in RPE cells and their response to recombinant SEMA3A further support the presence of a SEMA3A-responsive system in this lineage [15].

It remains unclear whether endogenous SEMA3A normally restrains RPE EMT. Recombinant protein may replace a regulatory signal that has weakened during disease, or it may exert an inhibitory effect that exceeds the physiological activity of an intact endogenous pathway. Intervention studies establish that PVR-related RPE responses remain sensitive to added ligand; they do not define the status of endogenous SEMA3A.

### 4.2. Evidence from Exogenous SEMA3A in PVR

Direct evidence comes principally from Bai et al. In ARPE-19 cells, recombinant SEMA3A reduced migration, induced cell-cycle arrest, and attenuated VEGF165-stimulated proliferation without suppressing basal proliferation. VEGF-associated phosphorylation of JNK and p38 MAPK was also partially reduced [15]. In the accompanying rabbit experiment, SEMA3A was administered together with ARPE-19 cells at model induction, and treated eyes developed less severe PVR over the following 14 days [15].

The common feature of the cellular and animal findings is an effect on the early migratory and proliferative phase of experimental PVR. Because SEMA3A was present from model induction, the study did not address delayed treatment of an established contractile membrane. The cellular work also covered only part of the RPE EMT phenotype. Junctional identity, ECM production, acquisition of contractility, and recovery of differentiated RPE features were not examined. Receptor expression was measured, but dependence on NRP1 or a specific plexin-A coreceptor was not tested. The study therefore supports early pharmacological activity of SEMA3A rather than an endogenous ligand deficiency as the cause of PVR.

### 4.3. Endogenous SEMA3A Expression in PVR

Transcriptomic data do not show a uniform reduction in endogenous *SEMA3A* transcript levels in PVR. In a mouse model of traumatic PVR, endogenous *Sema3a* transcript levels were significantly higher in RPE preparations collected seven days after injury than in controls, as reported in the authors’ published Source Data (adjusted *p* = 2.51 × 10^−6^) [10]. PVR was therefore present alongside increased endogenous *Sema3a* transcript abundance in this model.

In the human membrane dataset, *SEMA3A* transcript levels did not differ significantly between PVR membranes and internal limiting membrane (ILM) specimens (adjusted *p* = 0.278) [8]. PVR membranes contain RPE-lineage, immune, glial and myofibroblast-like cells. Bulk measurements reflect both expression within these populations and their relative abundance, potentially obscuring changes confined to RPE. The comparator was ILM rather than normal RPE; differences in species, injury setting and disease stage also limit comparison with the mouse data.

Recombinant SEMA3A reduced PVR severity when administered at induction in a separate rabbit study [15], but its efficacy does not demonstrate an endogenous protein deficit. It remains unclear whether higher *Sema3a* transcript levels lead to more biologically active protein, and whether any increase is sufficient to restrain fibrosis. The available evidence concerning exogenous and endogenous SEMA3A in PVR is summarized in Table 1.

Statistics are reproduced from the deposited human analysis and the mouse study’s published Source Data. No new differential-expression analysis was performed.

### 4.4. Post-Transcriptional Regulation and the miRNA Hypothesis

RNA abundance provides little information about how much SEMA3A protein is produced by an RPE cell undergoing EMT. miRNAs offer one mechanism by which protein output could fall without a proportionate reduction in steady-state mRNA. Their expression profiles change during RPE EMT [21,22,23], and Argonaute-containing silencing complexes can inhibit translation, promote mRNA decay, or do both.

Injury-induced transcription of *SEMA3A* may coincide with miRNA-mediated restriction of its translation. RNA abundance could then remain stable or increase without a comparable rise in protein. Enhanced transcription might also offset part of the miRNA-dependent RNA decay. Neither process can be inferred reliably from conventional mRNA sequencing, particularly when regulation is confined to a specific RPE state and diluted within a mixed surgical membrane.

No RPE or PVR study has yet linked a defined mature miRNA to endogenous *SEMA3A* target engagement, altered production of bioactive SEMA3A, and an EMT-related phenotype that can be rescued through the same target. Target prediction and inverse expression are useful for candidate selection, while reporter assays can verify sequence-dependent recognition. Evidence from these approaches remains preliminary unless the endogenous transcript and the resulting RPE phenotype are connected directly.

SEMA3A protein output may also be influenced by RNA-binding proteins, alternative 3′ untranslated region (3′ UTR usage), or stress-dependent changes in translation. Local pathway activity will further depend on the timing and cellular source of expression, protein secretion, extracellular stability, and receptor availability. Class 3 semaphorin processing and NRP1–plexin-A receptor assembly can modify signaling [16,17,18], although neither has been studied directly in PVR. Endogenous SEMA3A may also remain active but fail to counter the combined effects of inflammatory mediators, growth factors, and mechanical stress.

miRNA repression is especially relevant because it connects maintained RNA with altered protein output through a mechanism that can be tested at the endogenous target site. Recombinant SEMA3A bypasses intracellular translation and secretion, making its experimental activity compatible with this model. The result is not diagnostic, however; a pharmacological dose could also overcome restricted extracellular availability or stronger opposing signals.

### 4.5. Therapeutic Implications of Endogenous SEMA3A Regulation

The rabbit study does not by itself justify sustained intraocular supplementation with recombinant SEMA3A. Intraocular protein delivery would expose RPE, vascular, neural, and immune cells to the same ligand, although their responses to SEMA3A differ. Studies outside PVR have reported context-dependent effects in corneal fibroblasts, retinal vascular cells, neurons, and microglia [24,25,26,27,28,29]. Although these studies did not test the rabbit PVR regimen, they show that ocular responses to SEMA3A depend on cell type and disease context.

A regulatory defect confined to EMT-associated RPE cells would have different therapeutic implications. If such a defect is demonstrated and can be targeted selectively, correcting it could restore endogenous SEMA3A output within the relevant cellular state while reducing unnecessary exposure of the entire eye to recombinant protein. This possibility remains conditional on demonstrating that a defined miRNA–SEMA3A interaction is active in RPE cells and accounts for a relevant PVR phenotype. The immediate question is whether miRNAs already implicated in RPE EMT and PVR also regulate endogenous SEMA3A.

## 5. MicroRNAs at the RPE–PVR Interface

MicroRNAs help maintain the differentiated RPE phenotype and shape the response of RPE cells to EMT-inducing signals. In quiescent RPE, miRNA networks support epithelial identity, junctional organization, and cellular homeostasis. When RPE cells are exposed to TGF-β and other injury-associated cues, changes in these networks can influence the acquisition of migratory, matrix-producing, and contractile traits [21,22,23,30,31,32]. Functional studies support a causal contribution: manipulation of individual miRNAs alters RPE EMT phenotypes in vitro and, for a smaller group of candidates, modifies experimental PVR in vivo [33,34,35]. miRNAs therefore participate in the regulatory machinery that determines whether RPE cells retain their epithelial program or acquire properties that contribute to fibrocellular membrane formation.

### 5.1. MicroRNAs in RPE Differentiation and Homeostasis

The differentiated RPE has a characteristic miRNA repertoire. miR-204 and miR-211 are among its most abundant miRNAs and form part of an MITF-dependent program that maintains RPE differentiation, tight-junction organization, and transepithelial resistance. Increasing miR-204 and miR-211 promotes epithelial differentiation, whereas their inhibition disrupts RPE morphology and barrier function [30]. RPE-specific deletion of both miRNAs in adult mice subsequently produced RPE and retinal dysfunction, photoreceptor loss, and subretinal inflammation, providing in vivo evidence that this miRNA family is required for long-term RPE homeostasis [31]. miR-184 supports a related differentiation program by directly repressing AKT2 and reducing downstream mTOR activity [32].

The relevance to PVR lies in the starting state of the cell. RPE cells displaced through a retinal break are highly specialized epithelial cells rather than generic fibroblast precursors. Before contributing to a contractile membrane, they lose junctional organization, polarity, and tissue-specific functions. Reduced activity of miRNAs that normally stabilize the differentiated phenotype may make RPE cells more responsive to TGF-β, inflammatory mediators, serum components, and abnormal mechanical cues. miRNA dysregulation may therefore facilitate entry into EMT before a fully fibrotic phenotype has developed.

### 5.2. MicroRNA Control of Key RPE EMT Phenotypes

RPE EMT does not behave as a single molecular switch. Loss of epithelial identity, activation of the TGF-β transcriptional program, disruption of barrier function, migration, and acquisition of contractility are related but can be experimentally uncoupled. miRNAs have been implicated at each of these regulatory points.

At the signal-to-transcription interface, miR-1285 directly represses SMAD4 and attenuates the canonical TGF-β response [33]. miR-194 acts further downstream by targeting ZEB1, thereby limiting the loss of epithelial features and the development of a motile RPE phenotype [34]. let-7c has been associated with suppression of NF-κB-dependent responses, whereas miR-302d and miR-93 modify several components of the TGF-β and VEGFA programs [36,37,38]. These studies place miRNAs within the transcriptional machinery of RPE EMT, although pathway modulation should not be equated with direct binding to every affected transcript.

Other miRNAs act more clearly on the cell behaviors that form and contract a PVR membrane. In TGF-β1-treated RPE, miR-124 directly targets RHOG and reduces cytoskeletal remodeling, migration, and collagen-gel contraction [39]. miR-29b represses AKT2, with p65–Snail signaling operating downstream of this interaction [40,41]. miR-4516 acts through OTX1 and associated ERK signaling and affects migration and contraction in addition to EMT-marker expression [35]. These functional endpoints are especially relevant to PVR because membrane formation requires cell movement and traction, not simply a change in epithelial or mesenchymal markers.

The direction of a miRNA effect may change with the state of the RPE cell. In TGF-β-challenged primary human RPE, miR-302d suppressed EMT-associated changes and promoted partial recovery of epithelial morphology [38]. In a baseline differentiation model, however, miR-302d-3p directly targeted *CDKN1A*/p21 and promoted RPE dedifferentiation, proliferation, and migration [42]. miR-124 similarly suppressed EMT through RHOG in a TGF-β1 model but promoted migration and EMT-related changes through LIN7C under palmitate stress [39,43]. Work in high-glucose, palmitate, anti-VEGF, and epiretinal-membrane-related systems has identified further miRNA-dependent changes in RPE permeability, migration, and fibrotic signaling [44,45,46,47,48,49,50]. These models broaden the biological context but do not reproduce the inflammatory and mechanical environment of PVR.

A fixed classification of individual miRNAs as profibrotic or antifibrotic is therefore unlikely to be reliable. Interpretation depends on the mature strand, the differentiation state of the RPE cell, the targets available under that condition, and the phenotype that was measured. For PVR, the most informative studies are those that connect endogenous target regulation to migration, matrix remodeling, contraction, or an in vivo disease endpoint.

### 5.3. Evidence from Experimental PVR and Human Disease

Three miRNA mechanisms have been followed from cultured RPE into experimental PVR with relatively well-defined intracellular targets. miR-194 suppressed ZEB1-dependent EMT, migration, and contraction and reduced disease severity in a rat model [34]. miR-1285-overexpressing ARPE-19 cells showed a reduced capacity to induce PVR in rabbits, consistent with suppression of SMAD4-dependent signaling [33]. miR-4516 inhibited OTX1/ERK-associated migration and contraction and reduced rabbit PVR severity when intervention began at model induction [35]. These experiments connect miRNA–target regulation to a disease-level outcome, although they mainly alter the initiating RPE cells or intervene early. Reversal of an established contractile membrane has not been demonstrated.

Human PVR specimens add clinical relevance, although their mixed cellular composition complicates interpretation. miR-182 was lower in six PVR membranes than in primary RPE from healthy donors, whereas c-Met showed the opposite pattern. Restoring miR-182 directly repressed c-Met and reduced HGF-driven RPE proliferation and migration [51]. The functional relationship is persuasive, but comparison of a mixed fibrocellular membrane with isolated donor RPE cannot distinguish disease-associated regulation from differences in cellular composition. miR-29b expression in PVR membranes correlated positively with E-cadherin and inversely with α-SMA, complementing the AKT2–p65–Snail mechanism described in cultured RPE [40,41]. These observations remain cross-sectional and do not establish whether the miRNA change precedes membrane formation.

Extracellular miRNA studies have examined both intercellular signaling and potential ocular-fluid biomarkers. Small-EV preparations from TGF-β2-treated RPE can transfer experimentally enriched miRNA cargo and alter recipient-cell EMT responses [52,53]. Patient studies have identified hsa-miR-148a-3p, miR-21, and other candidate signals in vitreous or subretinal fluid from retinal detachment and proliferative vitreoretinal disease [54,55,56,57]. A paired serum–vitreous study confirmed that miRNA profiles differ between ocular and systemic compartments, but it did not include a PVR cohort [58]. Variability in patient selection, sampling, storage, assay platform, normalization, and extracellular-vesicle characterization continues to limit comparison between studies [59,60].

A biomarker need not identify its cell of origin to become clinically useful, whereas a claim about RPE-intrinsic SEMA3A regulation does. Fluid abundance and vesicular cargo cannot establish that the same miRNA is active within an RPE cell undergoing EMT. No ocular-fluid miRNA has yet undergone prospective analytical and clinical validation as a diagnostic or prognostic PVR biomarker.

The studies summarized here identify SMAD4, RHOG, AKT2, OTX1, and c-Met as miRNA targets in RPE cells [33,35,39,40,51]. They demonstrate effects on RPE phenotypes and, in selected models, experimental PVR, but do not establish that these effects are mediated by SEMA3A. Representative primary studies linking miRNAs to RPE identity, EMT, and PVR-related phenotypes are summarized in Table 2.

### 5.4. Current Evidence and Unresolved Questions

Current evidence is sufficient to establish miRNAs as regulators of differentiated RPE, EMT-associated behavior, and selected experimental PVR phenotypes. It does not identify a single dominant miRNA program in human PVR. The direction and magnitude of an effect vary with RPE differentiation, stimulus, target availability, and the disease endpoint examined.

Specific miRNAs have been shown to target SEMA3A in other cell types [61,62]. Together with the RPE findings summarized above, this evidence supports testing whether miRNA-mediated SEMA3A repression contributes to RPE EMT. Demonstrating this mechanism requires a defined mature miRNA to engage an endogenous *SEMA3A* site, alter biologically available SEMA3A protein, and produce an RPE phenotype that can be attributed to that interaction rather than to the miRNA’s other targets. No RPE or PVR study has yet completed this chain.

## 6. MicroRNA Regulation of SEMA3A: Evidence Beyond RPE and the Missing PVR Link

miRNAs regulate RPE EMT and several cellular behaviors that contribute to PVR, while exogenous SEMA3A modifies selected RPE responses associated with early disease. The molecular connection between these observations has not been demonstrated. To our knowledge, no study has shown endogenous miRNA–SEMA3A target engagement in RPE cells or established that a miRNA-dependent PVR phenotype is mediated by SEMA3A. Studies in other tissues are therefore useful as mechanistic precedents and sources of candidate miRNAs, but they cannot establish the retinal pathway.

### 6.1. miR-145-5p and miR-181b-3p as Complementary Mechanistic Precedents

Interest in miRNA-mediated regulation of SEMA3A during RPE EMT is not based on target prediction alone. Two extra-retinal studies provide complementary precedents for this hypothesis. The first shows that miR-145-5p can regulate human *SEMA3A* through defined sites in its 3′ untranslated region in activated fibroblast-like cells. The second places miR-181b-3p-mediated repression of SEMA3A within a TGF-β-driven mesenchymal transition and links it to cytoskeletal remodeling and tissue fibrosis. Neither study was performed in RPE cells or PVR, but together they establish that both the molecular interaction and its fibrotic consequences are biologically possible.

The miR-145-5p evidence arose from fibroblast-like synoviocytes isolated from patients with rheumatoid arthritis. These cells expressed more miR-145-5p and less SEMA3A than osteoarthritis controls, and inhibition of miR-145-5p increased endogenous *SEMA3A* mRNA. TGF-β also increased miR-145-5p expression in synovial fibroblasts [62]. Reporter experiments spanning the human *SEMA3A* 3′ UTR identified two responsive sites: mutation of either site reduced miR-145-5p-mediated repression, whereas mutation of both produced a greater loss of responsiveness. Recombinant SEMA3A also opposed VEGF165-induced survival, migration, and invasion in the same cellular setting [62]. The parallel with PVR is functional rather than lineage-based. RPE cells displaced during retinal detachment lose features of their differentiated state, become migratory, and respond to a growth-factor-rich environment that includes TGF-β and VEGF-related signals. These findings show that miR-145-5p can regulate SEMA3A and that recombinant SEMA3A can suppress fibroblast migration and invasion. They do not establish that miR-145-5p alters these behaviors through endogenous SEMA3A or that the interaction operates during epithelial EMT. Hong et al. used the designation miR-145; the reagent corresponds to hsa-miR-145-5p under current nomenclature. In human peripheral blood mononuclear cells (PBMCs), a miR-145-5p mimic reduced *SEMA3A* mRNA levels and the concentration of SEMA3A protein in culture supernatants [63]. In human jaw bone marrow mesenchymal stem cells, miR-145 inhibition promoted osteogenic differentiation, an effect partly attenuated by SEMA3A knockdown [64]. Together with the synovial fibroblast findings, these results support SEMA3A as a miR-145 target and indicate that this regulation can influence cell function.

The miR-181b-3p study provides a complementary fibrosis-oriented precedent. In human atrial endocardial endothelial cells, TGF-β/SMAD3 signaling increased miR-181b-3p while reducing SEMA3A and inducing endothelial-to-mesenchymal transition (EndMT). The SEMA3A-binding arm was identified as miR-181b-3p. Wild-type and mutant 3′ UTR reporters supported sequence-dependent targeting, and an endogenous target-site blocker restored *SEMA3A* mRNA and SEMA3A protein after exposure to TGF-β or a miR-181b-3p mimic. Inhibition of miR-181b-3p restored SEMA3A and reduced mesenchymal markers, whereas SEMA3A knockdown reproduced components of the transition. SEMA3A perturbation also linked this regulatory axis to LIMK/cofilin-associated actin remodeling. In TGF-β-transgenic mice, miR-181b-3p inhibition and recombinant SEMA3A each attenuated atrial fibrosis in parallel intervention arms [61]. This work provides a mechanistic sequence—from TGF-β induction of a mature miRNA to site-specific SEMA3A repression, cytoskeletal change, mesenchymal transition, and fibrosis—that closely resembles the regulatory problem posed by RPE EMT. It did not include AGO2-RIP or formal epistasis showing that loss of SEMA3A abolished the benefit of miR-181b-3p inhibition.

The miR-145-5p study establishes SEMA3A regulation in synovial fibroblasts, whereas the miR-181b-3p study places this regulation within TGF-β-induced EndMT and fibrosis. They do not identify either miRNA as the upstream regulator of SEMA3A in PVR. Indeed, a retinal endothelial study identified TMOD3, rather than SEMA3A, as the functional miR-145 target and did not confirm repression of the predicted *Sema3a* reporter [65], illustrating the importance of cellular context and target-site accessibility. The relevant question is whether either mature miRNA—or another miRNA expressed during RPE EMT—engages the endogenous *SEMA3A* transcript in the RPE state that contributes to PVR. Resolving this question requires arm-specific expression, confirmation of the *SEMA3A* 3′ UTR isoform used by RPE cells, endogenous target-site engagement, and a SEMA3A-dependent rescue of the RPE phenotype.

### 6.2. Other Reported miRNA–SEMA3A Relationships

Studies in vascular, renal, immune, skeletal, cancer, cardiac, and neural models show that SEMA3A can be regulated by several miRNAs (Table 3) [66,67,68,69,70,71,72,73,74,75,76]. Their relevance to PVR varies considerably. miR-15a-5p, for example, directly repressed SEMA3A during vascular smooth-muscle cell phenotypic switching and affected plaque stability in vivo [67]. This provides evidence that a miRNA–SEMA3A interaction can participate in cell-state remodeling, but the target landscape of vascular smooth-muscle cells differs from that of RPE.

Luciferase reporter assays using wild-type and mutant *SEMA3A* 3′ UTR constructs, together with AGO2-RIP and measurements of endogenous SEMA3A expression, support targeting by miR-16-5p and miR-32-5p [69,70]. These studies examined high-glucose-induced podocyte injury and EMT, migration, and invasion in oral squamous cell carcinoma cells, respectively. In both studies, phenotypic rescue involved combined manipulation of an upstream lncRNA (*TCF7* or *NR2F2-AS1*) and SEMA3A, rather than of the miRNA and SEMA3A. SEMA3A targeting has also been reported for miR-379-5p and miR-30b. Their functional studies concern processes distinct from RPE EMT: SEMA3A-dependent T-cell responses and uveitis for miR-379-5p [73], and axon growth and neuronal survival for miR-30b [74,75].

The remaining reports identify additional miRNAs that regulate SEMA3A but provide less direct guidance for PVR. miR-223-5p, previously designated miR-223*, has been linked to mouse *Sema3a* in septic cardiac injury [66]. miR-15b-5p and miR-23b-3p regulate SEMA3A in high-glucose podocyte models [68,72], whereas miR-196b-5p and miR-135b have been examined in skeletal or vascular RNA-network settings [71,76]. These studies show that SEMA3A is regulated by different miRNAs in several cell types.

miR-145-5p is induced by TGF-β in synovial fibroblasts; other studies demonstrate SEMA3A repression in PBMCs and a partly SEMA3A-dependent effect on cell function [62,63,64]. For miR-181b-3p, endogenous SEMA3A targeting has been demonstrated in a TGF-β-driven EndMT model, with miRNA inhibition also reducing atrial fibrosis [61]. We therefore focus on miR-145-5p and miR-181b-3p when considering whether TGF-β-induced miRNAs could suppress SEMA3A production and alter the RPE phenotype.

Studies summarized in Table 3 were conducted in models other than RPE/PVR. The miR-145 reagent used by Hong et al. [62] corresponds to hsa-miR-145-5p under current nomenclature; miRNA designations in [64,74,75] are retained as reported. Abbreviations: AGO2-RIP, Argonaute 2 RNA immunoprecipitation; EMT, epithelial–mesenchymal transition; EndMT, endothelial-to-mesenchymal transition; lncRNA, long non-coding RNA; miRNA, microRNA; PBMCs, peripheral blood mononuclear cells; PVR, proliferative vitreoretinopathy; RPE, retinal pigment epithelium; SEMA3A, semaphorin 3A; TGF-β, transforming growth factor-β; Th17, T helper 17 cells; UTR, untranslated region; WT/MUT, wild-type/mutant.

At present, miRNA regulation of SEMA3A is a well-supported and experimentally resolvable hypothesis, rather than an established retinal pathway. Its strength lies in the ability to test one endogenous interaction and follow its consequences from SEMA3A protein output to RPE behavior and experimental PVR. Figure 2 summarizes the available evidence and the proposed miRNA–SEMA3A relationship in RPE.

## 7. From Extra-Retinal Evidence to a Retinal Mechanism

The studies discussed in Section 6 show that SEMA3A can be regulated by miRNAs in several biological settings. Whether the same control operates in RPE during PVR remains unknown. In the retina, the relevant question is whether a mature miRNA expressed by transitioning RPE cells engages endogenous *SEMA3A*, reduces the ligand available for signaling, and thereby contributes to the cellular behavior that produces a contractile membrane.

### 7.1. Demonstrating Endogenous miRNA–SEMA3A Targeting in RPE

The miR-145-5p and miR-181b-3p studies provide useful precedents because they place SEMA3A under miRNA control in human cells and in disease settings involving migration, phenotypic transition, or fibrosis. Neither study identifies the miRNA–SEMA3A interaction used by RPE. Candidate selection in the retina should therefore begin with the exact mature strand expressed in human RPE, its temporal behavior during EMT, and the *SEMA3A* 3′ UTR isoform present in the same cells [61,62].

A sequence-dependent reporter response would identify a compatible recognition site, while AGO2-RIP could show that endogenous *SEMA3A* enters the RISC complex with the candidate miRNA. These assays address different questions and are not interchangeable. Reporter assays do not demonstrate use of the endogenous transcript, whereas AGO2 enrichment does not identify the binding site responsible. Protection or editing of the native *SEMA3A* site would provide more direct evidence that the interaction occurs in RPE and is required for repression.

Target engagement must also be connected to the phenotype. Expression of *SEMA3A* without the regulated 3′ UTR should counter the effects of a pathogenic miRNA, whereas SEMA3A depletion or neutralization should diminish the benefit of miRNA inhibition. Measurements should extend beyond *SEMA3A* mRNA to cellular and secreted protein, accompanied where possible by a receptor-dependent functional readout. This sequence distinguishes a miRNA that can repress a cloned *SEMA3A* reporter from one that materially limits endogenous SEMA3A during RPE EMT.

Reduced SEMA3A protein levels are not specific to miRNA-mediated repression. Other mechanisms that suppress translation or accelerate intracellular protein degradation could produce similar changes. Protein abundance alone does not establish biological activity, which can be altered by proteolytic processing [77]. Impaired secretion could also reduce extracellular SEMA3A despite continued protein synthesis.

Once released, SEMA3A may bind to the extracellular matrix, altering its distribution and access to responsive cells without necessarily losing biological activity [78]. Accelerated extracellular degradation could instead shorten the persistence of active SEMA3A even if secretion remains unchanged. At the receiving cell, reduced surface receptor availability, defective NRP1–plexin assembly or impaired downstream signaling could limit the response to biologically active SEMA3A [16,17]. Alternatively, endogenous SEMA3A signaling may remain functional yet be insufficient to counter the combined effects of growth factors, inflammation and mechanical stress in PVR.

Recombinant SEMA3A can bypass defects in synthesis or secretion and may temporarily compensate for limited extracellular availability. It may therefore overcome more than one limitation at the same time, so a beneficial response does not identify the underlying endogenous mechanism. Table 4 compares these possibilities, their predicted findings and the key experiments that could distinguish them.

### 7.2. Defining the Relevant RPE State and Disease Window

The importance of a miRNA–SEMA3A interaction will depend on the state of the RPE cell in which it occurs. Mature RPE, recently detached RPE, TGF-β-responsive cells, and the myofibroblast-like populations found in established membranes do not share the same transcript usage, target availability, or RISC composition. Human PVR membranes provide direct access to diseased tissue but represent multicellular lesions collected after fibrosis has become clinically apparent. Single-cell, lineage-resolved, and spatial approaches can identify the RPE populations in which a candidate miRNA and *SEMA3A* are expressed [8,9,10]. They cannot, on their own, determine whether those transcripts yield sufficient extracellular ligand or signaling activity.

Time-resolved experiments are needed to relate the mature miRNA arm to SEMA3A protein and to the RPE phenotype within the same biological window. Junctional loss, migration, matrix production, and contraction overlap during PVR and should not be treated as a fixed sequence. A miRNA that affects early migration may be less important once a collagen-rich contractile membrane has formed. The relevant retinal mechanism is therefore defined not only by target binding, but also by the RPE state and disease stage in which that binding has functional consequences.

### 7.3. Establishing Disease Relevance and Ocular Safety

A target relationship established in cultured RPE must next be tested in vivo. Conservation of the relevant *SEMA3A* 3′ UTR site should be confirmed before an animal model is used to evaluate a human miRNA interaction. Lack of conservation would require a species-specific reagent or a model carrying the human target sequence.

Timing is equally important. In the principal SEMA3A PVR study, recombinant ligand was administered together with the model-inducing RPE cells [15]. The experiment therefore supports activity during disease induction, not treatment of an established contractile membrane. A delayed-treatment arm is required before therapeutic activity can be claimed. Disease assessment should include membrane formation, traction and retinal detachment, supported by histological evidence of RPE transition and matrix deposition rather than EMT-marker changes alone.

Retinal structure and function should be examined alongside the PVR endpoint. SEMA3A has cell- and context-dependent effects on vascular, neural, and immune compartments of the eye [24,25,26,27,28,29], and broad, non-cell-selective inhibition of a miRNA may release many targets unrelated to SEMA3A. RPE-directed delivery or protection of the individual *SEMA3A* target site would therefore provide a cleaner in vivo test than non-selective manipulation of the entire miRNA network. These experiments would establish whether the interaction contributes to PVR after disease initiation and whether it can be modified without compromising the surrounding retina.

## 8. Translational Implications

The experiments outlined in Section 7 are intended to establish whether a disease-regulated miRNA limits endogenous SEMA3A in a defined RPE state. If this causal chain is confirmed, inadequate SEMA3A activity would become a measurable regulatory defect rather than an inference drawn solely from the response to recombinant ligand. This distinction has direct clinical relevance. The rabbit study shows that the pathway remains pharmacologically responsive when SEMA3A is administered at PVR induction [15], but it cannot identify which eyes have impaired endogenous signaling or whether ligand replacement is the most appropriate way to correct it.

Ocular-fluid miRNA studies provide a starting point for identifying such eyes. Several miRNAs have been detected in vitreous or subretinal fluid and associated with retinal detachment or proliferative vitreoretinal disease [54,55,56,57,58,59]. These observations are currently descriptive because fluid abundance does not establish cellular origin or intracellular target engagement. Once an endogenous RPE miRNA–SEMA3A interaction has been demonstrated, the corresponding mature miRNA strand could be examined together with SEMA3A protein or pathway activity. Concordance between the ocular-fluid signal, the relevant RPE state, and reduced SEMA3A output would support a mechanism-linked biomarker rather than a general marker of retinal injury. Its greatest value may lie in identifying high-risk or early, RPE-dominant disease in which preservation of SEMA3A remains biologically relevant. The same molecular relationship could subsequently be evaluated for treatment selection and pharmacodynamic monitoring. Prospective studies would still need to show that it adds information to established clinical risk factors and is not simply a consequence of mixed-cell injury.

Mechanistic validation would also determine which form of treatment is biologically appropriate. Recombinant SEMA3A remains a rational option when reduced ligand output is the limiting defect and NRP1/plexin signaling is intact. By contrast, inhibition of an induced pathogenic miRNA may be preferable when the same miRNA suppresses SEMA3A and other targets that cooperate in RPE EMT. This broader network effect could be therapeutically useful, although it would also increase the likelihood of altering unrelated functions in RPE and neighboring retinal cells. If the phenotype depends mainly on miRNA binding to a defined site in the *SEMA3A* 3′ UTR, target-site protection would offer a narrower approach by restoring endogenous SEMA3A without disabling the remainder of the miRNA network [61]. Such protection is interaction-selective rather than inherently RPE-selective, and its value depends on demonstrating that the endogenous site makes a material contribution to the phenotype. Studying upstream regulation therefore does not compete with ligand replacement; it determines whether the defect is best corrected by supplying SEMA3A, inhibiting a broader miRNA program, or selectively relieving repression of SEMA3A itself.

The likely clinical position of these strategies is also shaped by disease stage. Recombinant SEMA3A and individual miRNA interventions have been evaluated in separate studies, predominantly at or near PVR induction [15,33,34,35]. Their most plausible initial use would therefore be perioperative prevention or early disease modification in molecularly selected high-risk eyes, rather than pharmacological reversal of a mature contractile membrane. Established PVR would continue to require surgery, although pathway-directed treatment might reduce further membrane formation or postoperative recurrence. Intravitreal administration offers local access but not RPE specificity, and vitrectomy or tamponade may alter ocular exposure [81,82]. Retinal neural, vascular, and immune safety must also be preserved because both SEMA3A and individual miRNAs act outside RPE [24,25,26,27,28,29].

The miRNA–SEMA3A axis will acquire clinical value when the same molecular defect can identify a treatable group of eyes and guide an intervention that restores SEMA3A activity at a relevant disease stage without compromising retinal function. This is the translational consequence of establishing the upstream mechanism.

## 9. Conclusions

The available evidence does not support a simple model in which PVR progresses because endogenous *SEMA3A* transcript levels decline. Recombinant SEMA3A suppresses selected RPE responses and modifies experimental disease when present at model induction, whereas endogenous *Sema3a* transcript levels are significantly increased in RPE preparations from a mouse model of traumatic PVR. The unresolved issue is the adequacy of endogenous signaling: whether disease-relevant RPE states produce sufficient extracellular SEMA3A and whether the surrounding cells remain competent to respond. Pharmacological responsiveness to added ligand and possible impairment of endogenous pathway control should therefore be considered separately.

miRNA-mediated regulation could help explain this discrepancy. miRNAs regulate RPE differentiation, EMT-associated behavior, and experimental PVR, while extra-retinal studies show that SEMA3A can be directly controlled by specific miRNAs. The miR-145-5p and miR-181b-3p studies provide particularly relevant precedents for human target recognition, phenotypic transition, and fibrosis. These complementary lines of evidence have not yet been connected experimentally in RPE. The missing relationship is precise: a disease-regulated mature miRNA must be shown to restrict endogenous SEMA3A and thereby contribute to a PVR-relevant RPE phenotype.

From a precision-medicine perspective, the importance of this relationship lies in its potential to define a molecularly distinct form of PVR. An exact mature miRNA strand, considered together with SEMA3A protein or pathway activity, could identify an early RPE-dominant state in which endogenous signaling remains amenable to restoration. Such a signature would carry greater biological meaning than an isolated ocular-fluid association and could support molecular stratification, treatment selection, and pharmacodynamic monitoring.

Mechanistic validation would also determine how the pathway should be treated. Ligand replacement may remain appropriate when SEMA3A output is limiting and receptor signaling is intact. Inhibition of a pathogenic miRNA may be preferable when a broader profibrotic target network is involved, whereas protection of the endogenous *SEMA3A* target site could provide a more interaction-selective RNA strategy when that single regulatory edge is causal. Failure to confirm miRNA-dependent repression would redirect attention toward extracellular ligand availability, receptor competence, or other post-transcriptional mechanisms.

SEMA3A remains the disease-anchored question in this review; miRNA biology provides the route by which that question can be connected to molecular stratification and selective intervention. Establishing whether this regulatory axis operates in RPE would move the field from ocular-fluid association toward mechanism-linked biomarkers, and from empirical ligand supplementation toward therapy matched to a defined endogenous defect. This is the principal precision-medicine opportunity of the miRNA–SEMA3A axis in PVR.

## Figures and Tables

**Figure 1:**
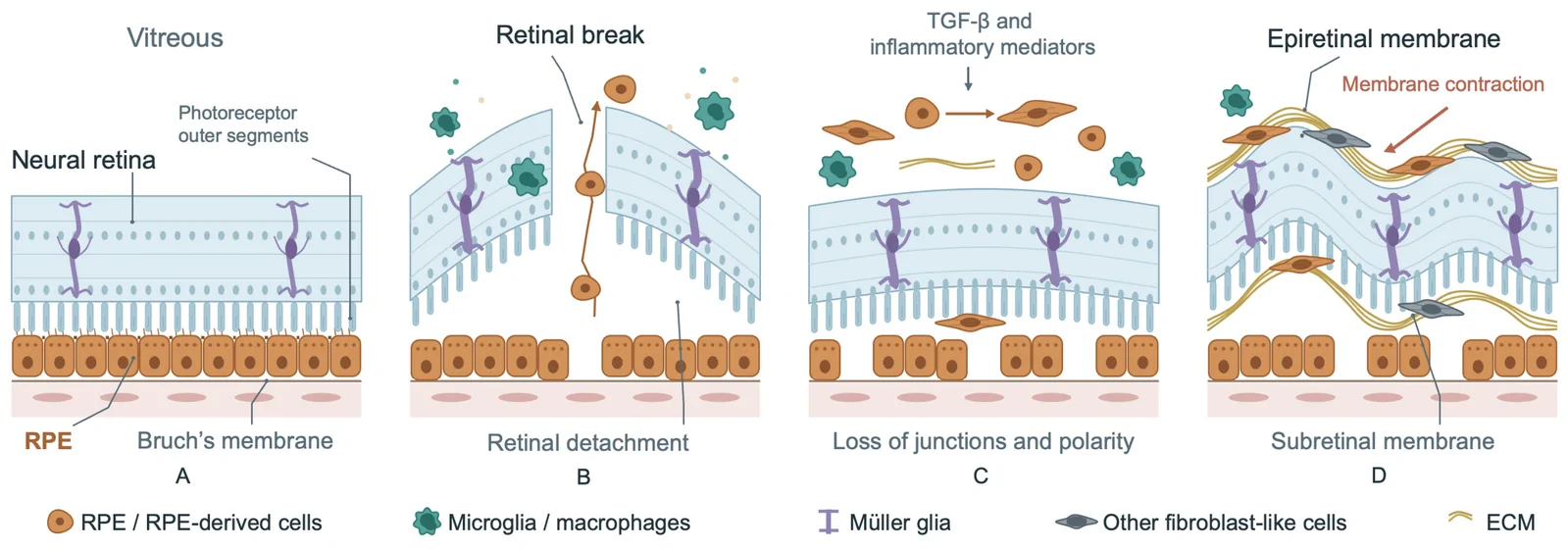
Cellular events underlying proliferative vitreoretinopathy. (**A**) In the attached retina, a polarized retinal pigment epithelial (RPE) monolayer lies beneath the photoreceptor outer segments. (**B**) Following a retinal break and detachment, RPE cells can leave their monolayer and enter the subretinal space or vitreous cavity, while inflammatory cells accumulate around the injured retina. (**C**) TGF-β and inflammatory mediators promote epithelial–mesenchymal transition (EMT) in RPE cells, with loss of junctions and polarity and acquisition of migratory, matrix-producing and contractile properties. Microglia/macrophages and reactive Müller glia contribute to the local inflammatory and fibrotic environment. (**D**) RPE-derived and other fibroblast-like cells contribute to epiretinal and subretinal fibrocellular membranes. Membrane contraction exerts traction on the retina, producing folds and potentially recurrent detachment. The illustrated processes overlap rather than occurring as discrete sequential stages [1,2,3,8,9]. ECM, extracellular matrix; PVR, proliferative vitreoretinopathy; TGF-β, transforming growth factor-β.

**Figure 2:**
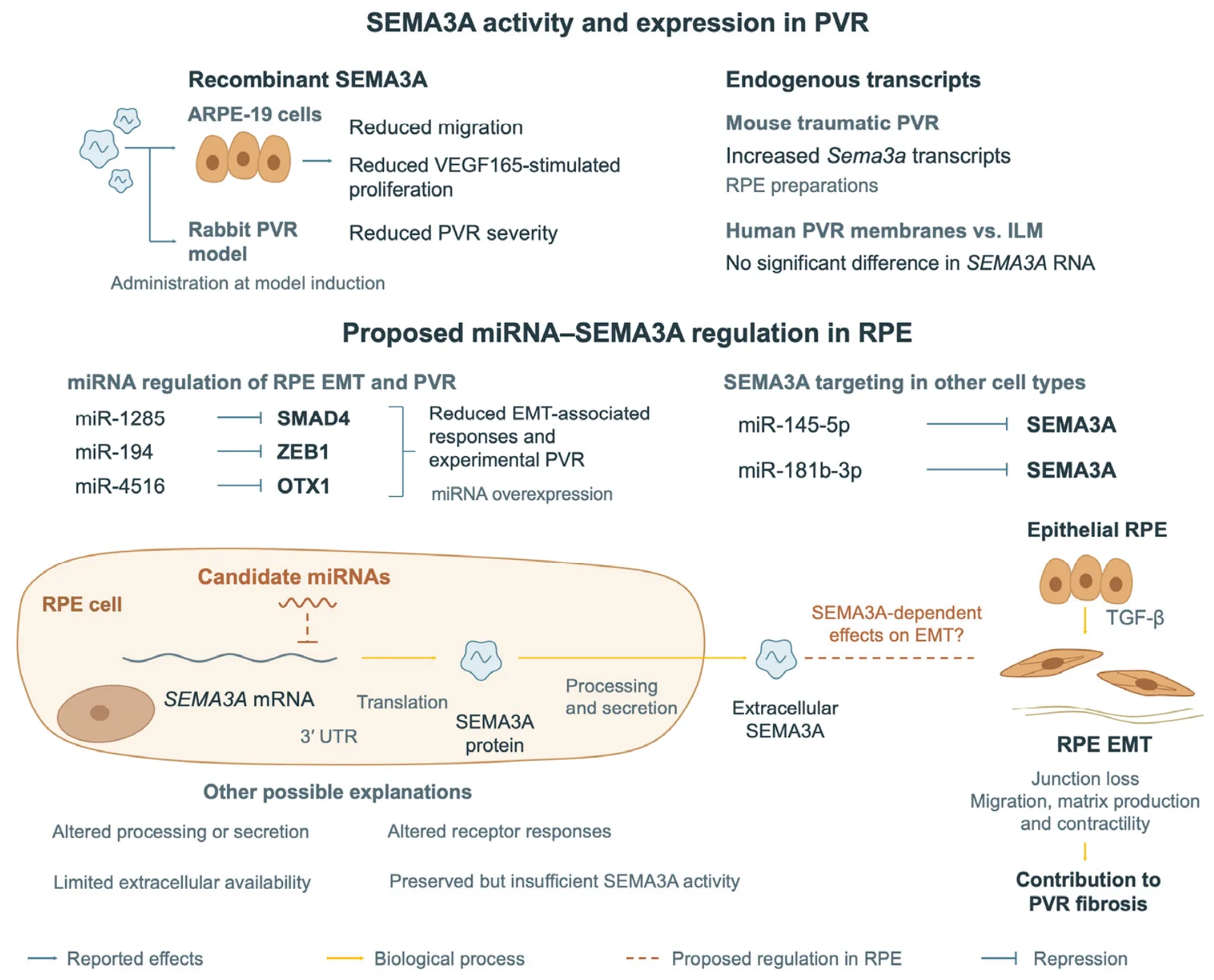
SEMA3A activity in PVR and the proposed role of miRNA regulation in RPE. Recombinant SEMA3A reduced ARPE-19 cell migration and VEGF165-stimulated proliferation and attenuated rabbit PVR when administered at model induction [15]. In separate transcriptomic studies, endogenous *Sema3a* transcripts were increased in RPE preparations from a mouse model of traumatic PVR, whereas *SEMA3A* RNA did not differ significantly between human PVR membranes and internal limiting membrane specimens [8,10]. These findings do not establish an endogenous SEMA3A protein deficit. Studies of miR-1285, miR-194 and miR-4516, targeting SMAD4, ZEB1 and OTX1, respectively, link miRNA regulation to RPE EMT-associated responses and experimental PVR [33,34,35]. Separate studies identify SEMA3A as a target of miR-145-5p and miR-181b-3p in other cell types [61,62]. The proposed mechanism connects miRNA-mediated repression of SEMA3A production to changes in RPE EMT, but this relationship remains to be established in RPE/PVR. Alternative explanations include altered processing or secretion, limited extracellular availability, altered receptor responses, and preserved but insufficient SEMA3A activity. Blue connections indicate reported experimental relationships; yellow arrows indicate biological processes; dashed orange connections indicate proposed regulation in RPE. Blunt-ended lines denote repression. EMT, epithelial–mesenchymal transition; ILM, internal limiting membrane; miRNA, microRNA; PVR, proliferative vitreoretinopathy; RPE, retinal pigment epithelium; SEMA3A, semaphorin 3A; TGF-β, transforming growth factor-β; UTR, untranslated region; VEGF, vascular endothelial growth factor.

**Table 1:** Available evidence concerning SEMA3A in PVR.

Evidence Source	Principal Observation	Interpretation
ARPE-19 experiments [15]	Recombinant SEMA3A reduced migration, induced cell-cycle arrest, and attenuated VEGF165-stimulated proliferation.	Selected early RPE responses are sensitive to added ligand; complete EMT, ECM production, and contraction were not examined.
Rabbit PVR model [15]	SEMA3A administered at model induction reduced PVR severity at day 14.	Supports prevention or early disease modification, not treatment of an established membrane.
Human PVR membranes, GSE179603 [8]	PVR–ILM comparison: log_2_ fold change, 1.016; adjusted *p* = 0.278.	No statistically significant difference in this comparison; bulk membrane data do not resolve RPE-specific expression.
RPE-enriched traumatic PVR, GSE244811/GSE244812 [10]	Day-7 RPE preparations: log_2_ fold change, 4.648; adjusted *p* = 2.51 × 10^−6^.	Significantly increased *Sema3a* RNA in this model; the corresponding protein response remains unresolved.

**Table 2:** Representative primary studies linking miRNAs to RPE identity, EMT, and PVR-related phenotypes.

miRNA	RPE/PVR Context	Mechanistic Link	Principal Phenotype	Evidence Boundary/Refs.
miR-204/miR-211	Differentiated human RPE; adult RPE-specific double knockout	MITF-linked identity program; adult RPE requirement	Differentiation, junctions, barrier, long-term homeostasis	Homeostatic evidence; no PVR intervention [30,31]
miR-184	Human RPE differentiation models	AKT2 direct; mTOR downstream	Promotes differentiation; limits proliferation/migration	Outside PVR [32]
miR-124	TGF-β1- or palmitate-treated RPE	RHOG or LIN7C direct, model dependent	Opposite effects on migration/EMT across models	Strong state dependence; no in vivo PVR [39,43]
miR-29b	TGF-β1-treated RPE; human PVR membranes	AKT2 direct; p65–Snail downstream	Reduces EMT-related signaling	Human evidence cross-sectional [40,41]
miR-194	TGF-β1-treated RPE; rat PVR	ZEB1 direct	Reduces EMT, migration, contraction, and PVR	Early experimental disease; limited human evidence [34]
miR-302d	Baseline and TGF-β-challenged RPE	*CDKN1A*/p21 direct at baseline; TGF-β/VEGFA-associated under challenge	Dedifferentiation at baseline; EMT suppression/reversion after TGF-β	Opposite state-dependent effects; no PVR endpoint [37,38,42]
miR-1285	TGF-β2-treated RPE; rabbit PVR	*SMAD4* reporter and endogenous modulation	Reduces EMT-related behavior and PVR-inducing activity	Initiating cells manipulated; not delayed therapy [33]
miR-4516	TGF-β2-treated RPE; rabbit PVR	OTX1 direct; ERK downstream	Reduces migration, contraction, and PVR	Intervention near induction; human relevance unknown [35]
miR-182	Human PVR membranes; cultured RPE	c-Met direct; AKT downstream	Limits HGF-driven proliferation/migration	Membrane-versus-donor-RPE comparison confounded [51]
miR-494	Human ERM discovery; RPE/Müller models	p27-mediated effect in RPE	Enhances TGF-β-responsive fibrotic change	ERM not PVR-specific; different sample matrices [48]

**Table 3:** Evidence for miRNA–SEMA3A regulation and its relevance to RPE EMT and PVR.

miRNA	Cell Model	SEMA3A Targeting and Expression	Phenotypic Effects	Relevance to RPE EMT/PVR
miR-145/miR-145-5p	Human synovial fibroblasts, PBMCs and jaw bone marrow mesenchymal stem cells [62,63,64]	WT/MUT 3′ UTR reporters; miRNA inhibition increased *SEMA3A* mRNA [62]. A miR-145-5p mimic reduced mRNA and supernatant protein in PBMCs [63].	miR-145 inhibition promoted osteogenic differentiation; SEMA3A knockdown partly attenuated this effect [64].	SEMA3A targeting in non-neoplastic cells and TGF-β induction of miR-145-5p provide a rationale for RPE EMT studies.
miR-181b-3p	Human atrial endocardial endothelial cells [61]	WT/MUT 3′ UTR reporters; the mimic reduced *SEMA3A* mRNA and SEMA3A protein, and target-site protection restored both.	miRNA inhibition reduced TGF-β-induced EndMT markers in vitro and attenuated atrial fibrosis in mice.	TGF-β-driven mesenchymal transition and fibrosis provide a functional precedent for RPE EMT/PVR studies.
miR-16-5p	High-glucose-treated human podocytes [69]	WT/MUT 3′ UTR reporters and AGO2-RIP; miRNA overexpression lowered, and inhibition increased, *SEMA3A* mRNA and SEMA3A protein.	lncRNA *TCF7* knockdown reduced oxidative stress, inflammation and apoptosis; SEMA3A re-expression counteracted these effects.	High-glucose-associated oxidative stress, apoptosis and inflammation rather than EMT.
miR-32-5p	Human oral squamous cell carcinoma cells [70]	WT/MUT 3′ UTR reporters and AGO2-RIP; miRNA inhibition increased *SEMA3A* mRNA and SEMA3A protein.	lncRNA *NR2F2-AS1* overexpression suppressed EMT, migration and invasion; SEMA3A knockdown reversed these effects.	EMT within a cancer-associated lncRNA network; its relevance to non-neoplastic RPE remains indirect.
miR-379-5p	Mouse CD4 + T cells [73]	3′ UTR targeting; miRNA negatively regulated SEMA3A expression.	miRNA inhibition reduced Th17 cells and ameliorated uveitis; SEMA3A knockdown counteracted these effects.	T-cell responses and ocular inflammation rather than EMT.
miR-30b	Rat retinal ganglion cells [74,75]	3′ UTR targeting and mRNA suppression [74]; miRNA overexpression lowered SEMA3A protein [74,75].	miRNA overexpression promoted axon growth [74,75] and reduced apoptosis [74].	Neuronal growth and survival rather than EMT.

**Table 4:** Potential mechanisms limiting endogenous SEMA3A activity: predicted findings and key mechanistic experiments.

Proposed Mechanism	Predicted Findings	Key Mechanistic Experiments
miRNA-mediated repression [61,62]	Reduced SEMA3A protein output, which may coexist with unchanged or increased transcript levels.	Protect or edit the endogenous miRNA-binding site; measure SEMA3A recovery and determine whether SEMA3A depletion attenuates any associated improvement in the RPE phenotype.
Altered SEMA3A processing [18,77]	Changes in SEMA3A molecular forms, with altered biological activity at comparable protein concentrations.	Identify cleavage products by immunoblotting or mass spectrometry; compare the biological activity of defined SEMA3A forms at matched molar concentrations.
Impaired SEMA3A secretion [79]	Reduced extracellular release despite continued intracellular synthesis.	Track newly synthesized SEMA3A in paired cellular and extracellular fractions by pulse–chase analysis, accounting for matrix binding and extracellular degradation.
Matrix-associated restriction of SEMA3A availability [78]	Increased matrix association with less SEMA3A reaching responsive cells, without necessarily reducing total protein abundance.	Quantify soluble and matrix-associated SEMA3A; alter matrix binding and assess RPE responses, using SEMA3A neutralization to test whether the effects depend on SEMA3A.
Reduced extracellular stability of SEMA3A [80]	Faster loss of intact, biologically active SEMA3A after release, despite unchanged secretion.	Add equal amounts of active SEMA3A to cell-free conditioned medium; measure intact protein and residual activity over time, with and without protease inhibitors.
Impaired NRP1–plexin signaling [16,17]	Reduced cellular responses despite adequate exposure to active SEMA3A; total NRP1 abundance may remain unchanged.	Measure surface NRP1 and plexin abundance, receptor-complex formation and downstream responses; test whether correcting the identified defect restores responsiveness to SEMA3A.
Preserved but insufficient endogenous activity	SEMA3A-dependent signaling remains detectable, but profibrotic RPE responses persist.	Deplete or neutralize endogenous SEMA3A during profibrotic stimulation and test for further increases in migration, matrix production or contraction.

Note. The cited studies provide the biological basis for the proposed mechanisms. The predicted findings and experimental approaches are the authors’ synthesis and have not been established in RPE/PVR. The mechanisms are not mutually exclusive.

## Data Availability

Public expression data and author-reported statistical results are available from the Gene Expression Omnibus under accessions GSE179603 and GSE244811/GSE244812 and from the published Source Data accompanying reference [10]. Extracted values, dataset details, and analytical notes are provided in Supplementary File S2. No new experimental data were generated.

## Supplementary Materials

The following supporting information can be downloaded at: https://www.mdpi.com/article/10.3390/biomedicines14102186/s1: Supplementary File S1, the literature search and study selection approach (Table S1); Supplementary File S2, summaries of studies supporting main-text Table 2 and Table 3, together with public gene-expression data and statistical results reported by the original investigators (Tables S2–S6).

## Abbreviations

AGO2, Argonaute 2; AGO2-RIP, Argonaute 2 RNA immunoprecipitation; ECM, extracellular matrix; EMT, epithelial–mesenchymal transition; EndMT, endothelial-to-mesenchymal transition; ERM, epiretinal membrane; EV, extracellular vesicle; ILM, internal limiting membrane; lncRNA, long non-coding RNA; miRNA, microRNA; NRP1, neuropilin-1; PBMCs, peripheral blood mononuclear cells; PVR, proliferative vitreoretinopathy; RISC, RNA-induced silencing complex; RPE, retinal pigment epithelium; RRD, rhegmatogenous retinal detachment; SEMA3A, semaphorin 3A; TGF-β, transforming growth factor beta; Th17, T helper 17; UTR, untranslated region; VEGF, vascular endothelial growth factor. WT/MUT, wild-type/mutant; α-SMA, α-smooth muscle actin.
