# Supplementary material for: SEMA3A in Proliferative Vitreoretinopathy: Exogenous Activity, Endogenous Regulation, and the MicroRNA Hypothesis
Source: Biomedicines. 2026 Sep 27;14(10):2186. doi: 10.3390/biomedicines14102186 (PMC13701372; doi:10.3390/biomedicines14102186)
Supplement: Supplementary file 1 [file biomedicines-14-02186-s001.zip › Supplementary File S1.pdf]

Supplementary File S1

Literature Search and Evidence-Selection Approach

This supplement documents the search and interpretive approach used for the focused narrative review. The review was not designed as a systematic review, and no PRISMA flow diagram, formal risk-of-bias instrument, or quantitative meta-analysis was applied.

Databases and date

PubMed/MEDLINE and Europe PMC were searched from database inception through 31 August 2026. Reference lists of eligible primary studies and recent reviews were also examined. Searches were updated on 31 August 2026 before finalizing the manuscript.

Table S1. Concept blocks

Concept	Representative terms
Disease	proliferative vitreoretinopathy OR PVR OR rhegmatogenous retinal detachment OR retinal detachment
Cell state	retinal pigment epithelium OR RPE AND epithelial mesenchymal transition OR EMT OR plasticity OR fibrosis
SEMA3A pathway	SEMA3A OR semaphorin 3A OR neuropilin-1 OR NRP1 OR plexin
Non-coding RNA	microRNA OR miRNA OR Argonaute OR RISC OR target-site blocker OR target protector
Translation	vitreous OR subretinal fluid OR extracellular vesicle OR biomarker OR oligonucleotide OR delivery OR precision medicine

Representative combined queries

- ("proliferative vitreoretinopathy" OR PVR) AND (SEMA3A OR "semaphorin 3A")
- ("retinal pigment epithelium" OR RPE) AND (EMT OR "epithelial mesenchymal transition") AND (microRNA OR miRNA)
- (SEMA3A OR "semaphorin 3A") AND (microRNA OR miRNA) AND (reporter OR AGO2 OR rescue OR "target-site blocker")
- (PVR OR "retinal detachment") AND (vitreous OR "subretinal fluid") AND (microRNA OR miRNA OR biomarker)
- (retina OR RPE) AND (oligonucleotide OR "RNA therapy" OR delivery) AND (intravitreal OR vitreous)

Selection and interpretation

- Priority was given to human PVR material, lineage-resolved or RPE-enriched models, well-characterized human RPE systems, sequence-specific target studies, rescue experiments, and in vivo PVR endpoints.
- Extra-retinal studies were included only when they tested a miRNA–SEMA3A relationship or provided a TGF-β-responsive fibrotic precedent relevant to the retinal question.
- Computational prediction, reporter activity, endogenous RISC engagement, native-site necessity, target-dependent rescue, and in vivo disease effects were treated as distinct forms of evidence rather than interchangeable proof.

- Ocular-fluid associations were interpreted as candidate biomarker evidence unless paired with source-resolved and mechanistic data.
- Intervention at model induction was distinguished from delayed treatment of established fibrosis.

Public expression datasets

Processed expression values and gene-level statistics were examined in the deposited analysis for GSE179603 and the published Source Data accompanying the mouse study. The mouse RNA-sequencing dataset, GSE244811, is a subseries of GSE244812. No new differential-expression analysis was performed. GSE179603 contains mixed, surgically excised human membrane specimens, whereas the mouse data derive from RPE preparations in a traumatic PVR model. Data sources, extracted values, reported statistics, and limitations are provided in Supplementary File S2.

Study summaries

Supplementary File S2 provides qualitative summaries of the studies supporting Tables 2 and 3 and documents the examination of the two public expression datasets. These summaries were used for narrative comparison; no formal evidence-grading system was applied.
