# Supplementary material for: SEMA3A in Proliferative Vitreoretinopathy: Exogenous Activity, Endogenous Regulation, and the MicroRNA Hypothesis
Source: Biomedicines. 2026 Sep 27;14(10):2186. doi: 10.3390/biomedicines14102186 (PMC13701372; doi:10.3390/biomedicines14102186)
Supplement: Supplementary file 1 [file biomedicines-14-02186-s001.zip › Supplementary File S2.pdf]

Supplementary File S2

Table S2. Representative miRNAs in RPE identity, EMT, and PVR

Selected studies supporting manuscript Table 2

miRNA	RPE/PVR context	Target/pathway	Principal phenotype	Disease proximity	Interpretive boundary
miR-204/miR-211 [30,31]	Differentiated human RPE; adult RPE-specific double knockout	MITF-linked identity program; adult RPE requirement	Differentiation, junctions, barrier, long-term homeostasis	RPE homeostasis	No PVR intervention
miR-184 [32]	Human RPE differentiation models	AKT2 direct; mTOR downstream	Differentiation; reduced proliferation/migration	RPE homeostasis	Outside PVR
miR-124 [39,43]	TGF-β1 or palmitate RPE	RHOG or LIN7C direct	Opposite migration/EMT effects across models	PVR-relevant in vitro	Strong state dependence; no in vivo PVR
miR-29b [40,41]	TGF-β1 RPE; human PVR membranes	AKT2 direct; p65–Snail downstream	Reduced EMT-related signaling	Human association + in vitro mechanism	Cross-sectional human evidence
miR-194 [34]	TGF-β1 RPE; rat PVR	ZEB1 direct	Reduced EMT, migration, contraction, PVR	Experimental PVR	Early model; limited human evidence
miR-302d [37,38,42]	Baseline and TGF-β-challenged RPE	CDKN1A/p21 direct at baseline; TGF-β/VEGFA-associated under challenge	Dedifferentiation at baseline; EMT suppression/reversion after TGF-β	RPE state biology	Opposite state-dependent effects; no PVR endpoint
miR-1285 [33]	TGF-β2 RPE; rabbit PVR	SMAD4 reporter and endogenous modulation	Reduced EMT-related behavior and PVR-inducing activity	Experimental PVR	Initiating cells manipulated; not delayed therapy
miR-4516 [35]	TGF-β2 RPE; rabbit PVR	OTX1 direct; ERK downstream	Reduced migration, contraction, PVR	Experimental PVR	Intervention near induction; human relevance unknown
miR-182 [51]	Human PVR membranes; cultured RPE	c-Met direct; AKT downstream	Reduced HGF-driven proliferation/migration	Human tissue + in vitro mechanism	Membrane-versus-donor-RPE comparison confounded
miR-494 [48]	Human ERM discovery; RPE/Müller models	p27-mediated effect in RPE	Enhanced TGF-β-responsive fibrotic change	Adjacent human membrane context	ERM not PVR-specific; different sample matrices

Note. These studies describe miRNA regulation of RPE homeostasis, EMT-associated responses, or experimental PVR. They do not establish that these effects are mediated by SEMA3A.

Table S3. Representative miRNA–SEMA3A relationships outside RPE/PVR

Mechanistic precedents selected for manuscript Table 3

miRNA	Model/context	Target-level evidence	Phenotype	Relevance to RPE EMT/PVR	Transfer limitation
miR-145/miR-145-5p [62–65]	Human synovial fibroblasts, PBMCs and jaw bone marrow mesenchymal stem cells	Wild-type and mutant 3′ UTR reporters; miRNA inhibition increased *SEMA3A* mRNA in synovial fibroblasts. A miR-145-5p mimic reduced *SEMA3A* mRNA and SEMA3A protein in PBMC culture supernatants.	miR-145 inhibition promoted osteogenic differentiation; SEMA3A knockdown partly attenuated this effect.	TGF-β induction of miR-145-5p in synovial fibroblasts and SEMA3A regulation in human cell models.	Partial target-dependent phenotypic rescue was demonstrated in osteogenic differentiation, not synovial fibroblast migration. A retinal endothelial study did not confirm repression of the *Sema3a* reporter.
miR-181b-3p [61]	Human atrial endocardial endothelial cells; TGF-β mouse model	Wild-type and mutant 3′ UTR reporters; the miRNA mimic reduced *SEMA3A* mRNA and SEMA3A protein, and endogenous target-site protection restored both.	miRNA inhibition reduced TGF-β-induced EndMT markers in vitro and attenuated atrial fibrosis in mice.	TGF-β transition + fibrosis	No AGO2-RIP or formal epistasis; not RPE
miR-16-5p [69]	High-glucose human podocytes	Wild-type and mutant 3′ UTR reporters and AGO2-RIP; miRNA overexpression lowered, and inhibition increased, *SEMA3A* mRNA and SEMA3A protein.	lncRNA TCF7 knockdown reduced oxidative stress, inflammation and apoptosis; SEMA3A re-expression counteracted these effects.	Oxidative stress, inflammation and apoptosis rather than EMT.	Renal context; no native-site necessity
miR-32-5p [70]	Human oral squamous carcinoma	Wild-type and mutant 3′ UTR reporters and AGO2-RIP; miRNA inhibition increased *SEMA3A* mRNA and SEMA3A protein.	lncRNA NR2F2-AS1 overexpression suppressed EMT, migration and invasion; SEMA3A knockdown reversed these effects.	EMT within a cancer-associated lncRNA network.	Cancer-associated ceRNA context
miR-379-5p [73]	Mouse CD4+ T cells; autoimmune uveitis	3′ UTR targeting; miRNA negatively regulated SEMA3A expression.	miRNA inhibition reduced Th17 cells and ameliorated uveitis; SEMA3A knockdown counteracted these effects.	T-cell responses and ocular inflammation rather than EMT.	Immune-cell regulation, not RPE-autonomous EMT.
miR-30b (arm unspecified in source) [74,75]	Rat RGCs; optic-nerve injury	Reported 3′ UTR targeting and mRNA suppression; miRNA overexpression lowered SEMA3A protein.	miRNA overexpression promoted axon growth and reduced apoptosis; another study examined retinal ganglion cell polarity.	Neuronal growth and survival rather than EMT.	Not RPE EMT; altering SEMA3A may affect retinal neurons

Note. *These studies examine miRNA–SEMA3A regulation in models other than RPE/PVR. They do not establish endogenous miRNA–SEMA3A target engagement in RPE cells or a SEMA3A-dependent effect on RPE EMT or PVR. The miR-145 reagent used by Hong et al. corresponds to hsa-miR-145-5p under current nomenclature. Other miRNA names are retained as reported where the mature arm has not been confirmed.*

Public-data inspection

Gene-level expression values and differential-expression statistics were extracted from the authors' publicly available processed data and Source Data files. No raw sequencing data were reanalysed, and no P values were recalculated for this review.

Table S4. Human surgical-membrane dataset (GSE179603)

Dataset	Source field	Reported value	Description	Source URL
GSE179603	PVR membrane mean	1489.55	Mean normalized reads in PVR membranes; n=10	https://www.ncbi.nlm.nih.gov/geo/query/acc.cgi?acc=GSE179603
GSE179603	Macular-pucker mean	1467.67	Mean normalized reads in macular-pucker membranes; n=10	https://www.ncbi.nlm.nih.gov/geo/query/acc.cgi?acc=GSE179603
GSE179603	ILM mean	695.07	Mean normalized reads in ILM specimens; n=7	https://www.ncbi.nlm.nih.gov/geo/query/acc.cgi?acc=GSE179603
GSE179603	log2FC_PVR_ILM	1.016	Reported $\log_2$ fold change for PVR versus ILM	https://www.ncbi.nlm.nih.gov/geo/query/acc.cgi?acc=GSE179603
GSE179603	padj_PVR_ILM	0.278	Reported adjusted P value for PVR versus ILM	https://www.ncbi.nlm.nih.gov/geo/query/acc.cgi?acc=GSE179603

Note. *Human SEMA3A expression data were obtained from the publicly available dataset accompanying the original study (GEO accession: GSE179603). Group means and comparison statistics are reproduced from the authors' analysis; the $\log_2$ fold change was not recalculated from the displayed means. The PVR-versus-ILM comparison did not show a statistically significant difference. These were mixed surgical specimens, and the ILM comparator was not normal RPE.*

Table S5. **RPE preparations from a mouse model of traumatic PVR (GSE244811, a subseries of GSE244812)**

Group	Sample identifier	Processed *Sema3a* expression value
Control	NC_1	1.0307
Control	NC_2	5.5566
Control	NC_3	0
PVR	PVR_1	30.8912
PVR	PVR_2	115.0349
PVR	PVR_3	20.7782

Table S6. **Differential-expression statistics reported in the original study**

Comparison	$\log_2$ fold change	Adjusted *P* value
PVR versus control	4.648	2.51×10^{-6}

Note. *Mouse Sema3a expression values and differential-expression statistics were obtained from the published Source Data accompanying the original study. The RNA-sequencing dataset is available under GEO accession GSE244811, a subseries of GSE244812. RPE preparations were collected seven days after PVR induction. Each group comprised three pooled biological replicates, with RPE cells from three animals contributing to each replicate. NC denotes the normal control group. Sample identifiers follow the Source Data, and expression values are presented on the supplied scale, rounded to four decimal places. The statistics are reproduced from the authors' analysis and indicate increased Sema3a transcript abundance in PVR compared with controls.*

No new differential-expression analysis was performed for this review. The human and mouse datasets were considered separately because they differ in species, injury setting, tissue composition and disease stage. Neither dataset establishes the amount, extracellular availability or biological activity of endogenous SEMA3A protein.
